# Parasite‐Derived IL‐33 Inhibitors Prevent Bleomycin‐Induced Lung Fibrosis

**DOI:** 10.1002/eji.70291

**Published:** 2026-10-08

**Authors:** Samuele Di Carmine, Tania Frangova, Suzanne H. Hodge, Danielle J. Smyth, Tera Birchall, Josh Richards, Ian Newton, Sarah Kelly, Alice R. Savage, E. Suzanne Cohen, J. Simon C. Arthur, Henry J. McSorley

**Affiliations:** ^1^ Division of Cell Signalling and Immunology School of Life Sciences University of Dundee Dundee UK; ^2^ Bioscience Asthma and Skin Immunity Research and Early Development Respiratory & Immunology BioPharmaceuticals R&D, AstraZeneca Cambridge UK

**Keywords:** *Heligmosomoides polygyrus bakeri*, Idiopathic pulmonary fibrosis, IL‐33 blockade, IL‐33, type 2 inflammation

## Abstract

Idiopathic pulmonary fibrosis (IPF) is a progressive lung‐scarring disease in which epithelial injury, consequent release of alarmins such as interleukin (IL)‐33, and immune activation contribute to extracellular matrix deposition. The intestinal helminth *Heligmosomoides polygyrus bakeri* (*Hpb*) secretes immunomodulators that suppress type 2 immunity, including HpARI2, which binds IL‐33, and HpBARI_Hom2, which blocks the IL‐33 receptor ST2. Here, we test this parasite‐derived dual blockade strategy in the bleomycin (BLM) mouse model, moving from live *Hpb* infection to administration of total *Hpb* excretory/secretory products (HES), and finally administering recombinant HpARI2 and HpBARI_Hom2 proteins. *Hpb* infection and HES treatment prevented BLM‐induced lung fibrosis when administered during the early inflammatory phase. Recombinant HpARI2+HpBARI_Hom2 reproduced this protection, reduced collagen deposition and hydroxyproline accumulation, and dampened ILC2, eosinophil, macrophage, and neutrophil responses. In contrast, HES or HpARI2+HpBARI_Hom2 administration failed to reverse established fibrosis when administered later in the model. Similarly, administration of the IL‐33trap fusion protein could replicate these effects, suppressing fibrosis when administered in the early inflammatory, but not later fibrotic phases of this model. These findings identify parasite‐derived inhibition of the IL‐33 axis as a defined molecular mechanism by which helminth products can prevent early inflammatory fibrogenesis, while also demonstrating that IL‐33 blockade is unlikely to reverse established lung scarring.

## Graphical abstract

Infection with the intestinal helminth *Heligmosomoides polygyrus bakeri*, or administration of the parasite products (HES), or administration of recombinant HpARI2+HpBARI_Hom2 can all suppress the development of lung fibrosis in the mouse bleomycin model, via suppression of the IL‐33 pathway. Graphic abstract created in BioRender. McSorley, HJ. (2026) https://BioRender.com/8ag7ll4.

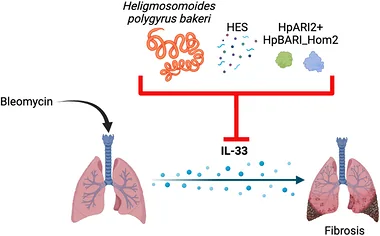

**Abbreviations**
AT2: alveolar type 2 epithelial cell
BALF: bronchoalveolar lavage fluid
BLM: bleomycin
BSA: bovine serum albumin
COPD: chronic obstructive pulmonary disease
ECM: extracellular matrix
FBS: foetal bovine serum
FFPE: formalin‐fixed paraffin‐embedded
GIT: gastrointestinal tract
HES: helminth excretory/secretory products
HpARI: *Hpb* alarmin release inhibitor
HpARI2: Hpb alarmin release inhibitor protein 2
*Hpb*
: *Heligmosomoides polygyrus bakeri*
HpBARI: *Hpb* binds alarmin receptor and inhibits protein
HpBARI_Hom2: *Hpb* binds alarmin receptor and inhibits protein 2
IL: interleukin
IL‐1RAcP: interleukin‐1 receptor accessory protein
ILC2: type 2 innate lymphoid cell
IPF: idiopathic pulmonary fibrosis
MAPK: mitogen‐activated protein kinase
MFI: mean fluorescence intensity
NF‐κB: nuclear factor kappa‐light‐chain‐enhancer of activated B cells
OD: optical density
PBS: phosphate‐buffered saline
ROI: region of interest
SD: standard deviation
sST2: soluble ST2
ST2: serum‐stimulation 2
TSLP: thymic stromal lymphopoietin
VAL: venom allergen‐like protein

## 1 Introduction

Idiopathic pulmonary fibrosis (IPF) is a chronic interstitial lung disease characterised by abnormal accumulation of extracellular matrix (ECM), destruction of alveolar architecture, and loss of lung function [1]. Despite intensive research, the precise mechanisms driving disease initiation and progression remain uncertain. However, environmental and occupational exposures to agents such as silica [2], wood [3], metal dust [4], and cigarette smoke [5] are IPF risk factors. These trigger damage to bronchial and alveolar epithelial cells, which could thereby release alarmin molecules into the extracellular milieu [6].

Among the many cellular and molecular players implicated in IPF, IL‐33, a member of the IL‐1 family of cytokines, has emerged as a key mediator linking epithelial injury, immune activation, and fibroblast function during IPF. IL‐33 is constitutively expressed by alveolar type 2 (AT2) epithelial cells in the lung, where it is stored in the nucleus until epithelial damage and necrosis cause its release [7]. Upon release, IL‐33 signals through the ST2 receptor (IL1RL1) and its co‐receptor IL‐1RAcP, activating downstream pathways including NF‐κB and MAPK, which lead to the activation and proliferation of immune cells [8]. In IPF, IL‐33 expression is upregulated in the lungs of asthma, COPD, and IPF patients [9, 10], and elevated IL‐33 levels have been correlated with progressive tissue injury and disease severity [10, 11, 12]. IL‐33 contributes to fibrosis by promoting type 2 immune responses, characterised by increased production of IL‐4, IL‐5, and IL‐13, which favour alternative macrophage activation and eosinophil recruitment, both of which are known to support fibrotic remodelling [11, 13, 14].

Targeting the IL‐33/ST2 axis represents a promising approach for intervention in IPF. In mouse models, studies in which the IL‐33 pathway is blocked, either through genetic deletion (e.g., IL‐33 or ST2 knockout mice) or pharmacologic inhibition (e.g., anti‐IL‐33 or anti‐ST2 antibodies administered throughout the model), have consistently shown attenuation of fibrosis when the pathway is blocked from the beginning of the model [11, 15, 16, 17, 18]. These interventions dampen the type 2 immune environment that drives fibrogenesis. However, because these models involve blocking IL‐33 signalling from the outset, it remains unclear whether IL‐33 inhibition is solely preventative or if it can also act therapeutically once fibrosis is established.

Addressing this distinction was the goal of the current study. Although approved anti‐fibrotic agents such as Nintedanib [19] and Pirfenidone [20] can slow disease progression, they do not reverse established fibrosis, nor do they target the upstream immune mechanisms contributing to disease initiation and progression. A new approach to fibrosis prevention could be to utilise pathogen‐derived immunomodulation. Helminths provide a biologically distinct route into this question because they have evolved secreted proteins that modulate host alarmin responses. In this regard, the immune‐regulatory properties of the helminth *Heligmosomoides polygyrus bakeri* (*Hpb*), particularly through its secreted products, offer an exciting avenue for therapeutic development [21]. *Hpb* can suppress liver and skin fibrosis through poorly understood mechanisms [22, 23]. *Hpb* secretes a complex mixture of immunomodulatory molecules known as excretory‐secretory products (HES), which can reproduce the anti‐inflammatory effects of live infection [24, 25]. HES consists of over 370 proteins, including apyrases, proteases, acetylcholinesterases, and venom allergen‐like (VAL) proteins, many of which are stage‐specific and may play roles in immune evasion [21, 26]. Of particular interest are two key immunomodulatory families: the *Hpb* alarmin release inhibitor proteins (HpARI1‐3) [27, 28] and the binds alarmin receptor and inhibits proteins (HpBARI and HpBARI_Hom2) [29]. HpARI1 and HpARI2 suppress IL‐33 signalling by binding IL‐33 directly, blocking its interaction with its receptor ST2 [28], while HpBARI and HpBARI_Hom2 bind and block ST2 [29]. HpBARI_Hom2, in particular, has the highest affinity for human ST2 and effectively blocks human IL‐33 signalling, making it a potential candidate for therapeutic development [29]. Thus, HpARI2 and HpBARI_Hom2 provide a parasite‐derived means to block both ligand and receptor within the same alarmin pathway.

We therefore used the BLM model to determine whether parasite‐derived molecules can alter lung fibrosis, and whether dual ligand/receptor blockade of IL‐33 signalling is sufficient to explain this effect. The study was structured as a stepwise test of mechanism: first assessing live *Hpb* infection, then total HES, then recombinant HpARI2+HpBARI_Hom2, followed by comparison with an engineered IL‐33trap protein [30]. We also varied the timing of treatment to distinguish prevention during inflammatory initiation from reversal of established fibrotic pathology.

Our results show that *Hpb* prevents BLM‐induced fibrosis, and this effect can be replicated by administration of HES, HpARI2+HpBARI_Hom2, or IL‐33trap. However, administering HES, HpARI+HpBARI_Hom2, or the IL‐33trap at the late phase of the model could not reverse established fibrosis. This confirms the role of IL‐33 in initiating lung fibrosis, but halting its signalling cannot reverse established lung scarring.

## 2 Materials and Methods

### 2.1 Mice and Experimental Models

All procedures conformed to the University of Dundee guidelines and the UK Animals (Scientific Procedures) Act 1986 (Project License PP9520011). Female C57BL/6J mice (5 weeks old) were obtained from Charles River, UK, and acclimatised for 10 days. Female mice only were used, as our data suggested that males were more susceptible to BLM‐induced pathology, and increased numbers reached severity limits. Mice (>7 weeks) were ear‐notched for identification and health‐checked before use. Animals were cage‐blocked, with one mouse per group per cage to minimise bias.

### 2.2 Recombinant Protein Expression and Purification

Plasmids encoding HpARI2 [29], HpBARI_Hom2 [27], IL‐33trap [30], and TSLPtrap [31], all with 6His tags for purification, were amplified in *E. coli* DH5α and purified using Hi Pure Midi kits (Thermo Fisher). Proteins were expressed in mammalian systems and purified via HisTrap Excel and nickel‐charged HP Chelating columns (Cytiva). Final preparations were dialysed in PBS, sterilised by 0.22 µm filtration, quantified by NanoDrop, and stored at −20°C. Using the HEKBlue TLR4 reporter cell line (Invivogen), all proteins were found to be free of endotoxin contamination.

### 2.3 The Bleomycin Model

Pulmonary fibrosis was induced via a single oropharyngeal dose of bleomycin (BLM, 1.5 mg/kg in 50 µL sterile PBS; Sigma‐Aldrich) under isoflurane anaesthesia. Mice were either culled at day 7 (inflammatory phase) or day 21 (fibrotic phase).

### 2.4 Murine Infection With *Hpb*

Mice were orally gavaged with 200 L3 *Hpb* larvae in 200 µL distilled water either 28 or 7 days before BLM challenge. Weights were recorded pre‐infection and pre‐BLM to monitor health.

### 2.5 HES and Recombinant Protein Administration

Following BLM administration, mice were treated with 3 intraperitoneal doses of HES or recombinant proteins (10 µg HES, 10 µg HpARI2 + 10 µg HpBARI_Hom2; 20 µg IL‐33 trap; or 50 µg TSLP trap in 100 µL PBS) every 2 days during weeks 1, 2 or 3 post‐BLM.

The IL‐33trap used in this study is a fusion protein comprising the extracellular domains of mouse ST2 and IL‐1RAcP, fused to a human IgG1 Fc tag. This design enables high‐affinity binding to IL‐33, while the Fc portion enhances protein stability and extends in vivo half‐life [30]. The TSLPtrap is a fusion protein including the ectodomains of the TSLPR and IL‐7 receptor [31]. Together, the ectodomains of TSLPR and IL‐7Rα form a heterodimeric receptor complex that binds TSLP with high affinity [32].

### 2.6 Tissue and Sample Harvesting

At endpoints (days 7 or 21 post‐BLM), mice were euthanised under terminal anaesthesia (1:1 mix of 100 mg/mL medetomidine hydrochloric acid (HCl) and 1 mg/mL ketamine HCl) via exsanguination. Bronchoalveolar lavage fluid (BALF) was collected using 4 × 0.5 mL cold PBS washes. Lung lobes were processed for histology (left lobe), flow cytometry (superior/middle lobes), or snap‐frozen for hydroxyproline quantification (inferior/post‐caval lobes).

Where material was available, day 21 BALF supernatants and snap‐frozen lung tissue were also assessed by exploratory cytokine ELISA and qPCR analyses.

### 2.7 Formalin‐Fixed Paraffin Embedded (FFPE)

Left lung lobes were fixed in 10% formalin (∼24 h), transferred to 70% ethanol, and embedded in paraffin. Sections (5 µm thick) were cut using a rotary microtome (Thermo Fisher), mounted on Superfast slides (Thermo Fisher) and air‐dried overnight before dewaxing and staining.

### 2.8 BALF and Lung Cell Flow Cytometry

Lungs were digested in Liberase TL (2 U/mL) (Sigma‐Aldrich) and DNase I (80 U/mL) (Thermo Fisher) for 35 min at 37°C. Enzymes were inactivated with complete RPMI (10% FBS, 1% pen‐strep, 1% L‐glutamine) (all from Gibco, Thermo Fisher). Cells were filtered through a 70 µm strainer (Greiner Bio‐One, Austria), red blood cell‐lysed with ACK buffer (Thermo Fisher), counted, and resuspended in cRPMI at 1 × 10^6^ cell/mL for flow cytometry. Lung single‐cell suspensions or cells isolated from BALF (all 1 × 10^6^ cells) were stained with 200 µL/sample of Fixable Blue Live/Dead stain (1:1000 in 1X PBS, Thermo Fisher) at 4°C for 20 min in the dark. Cells were spun down and washed once with 1X PBS and once with fluorescence‐activated cell sorting (FACS) buffer [1X PBS with 0.5% Bovine serum albumin (BSA) (Sigma‐Aldrich) and 0.05% sodium azide (Thistle Scientific, Glasgow, UK)]. Cells were then blocked with α‐mouse CD16/32 antibody (1:50 in FACS buffer, Biolegend, California, USA) at 4°C for 10 min. Cells were washed with FACS buffer and surface‐stained using antibodies against the following targets, incubating at 4°C for 20 min: CD3ε (clone: 145‐2C11, Biolegend), CD4 (clone: RM4‐5, Biolegend), CD5 (clone: 53‐7.3, Biolegend), CD11b (clone: M1/70, Biolegend), CD19 (clone: 6D5, Biolegend), CD25 (clone: PC61.5, Biolegend), CD45 (clone: 30‐F11, Biolegend), CD49b (clone: DX5, eBioscience), GR1 (clone: RB6‐8C5, Biolegend), CD278 (ICOS) (clone: C398.4A, Biolegend), KLRG1 (clone: 2F1/KLRG1, Biolegend), ST2 (clone: RMST2‐2, eBioscience), CD11b (clone: M1/70, Biolegend), CD11c (clone: N418, Biolegend), CD45 (clone: 30‐F11, Biolegend), CD206 (clone: C068C2, Biolegend), F4/80 (clone: BM8, Biolegend), Ly‐6G (clone: 1A8, Biolegend), SiglecF (clone: REA798, Miltenyi Biotec). Cells were rewashed with FACS buffer and then resuspended in 200 µl of FACS buffer. Samples were filtered before acquisition through a 70 µm mesh and acquired on an LSR Fortessa (BD Biosciences, Haryana, India). Data analysis was performed on FlowJo v10 (Treestar, Oregon, USA). For gating strategies, refer to Supporting Information Figures.

Gating was performed on live singlets. For the lymphoid panel, lineage‐positive cells were excluded using the FITC dump channel (CD3e, CD5, CD11b, CD19, CD49b, and GR‐1), and ILC2s were identified as CD45^+^lineage^–^CD4^–^ICOS^+^CD25^+^ cells. For the myeloid panel, eosinophils were identified as CD45^+^CD11b^+^Siglec‐F^+^ cells, alveolar macrophages as CD45^+^CD11c^+^Siglec‐F^+^F4/80^+^ cells, and neutrophils as CD45^+^CD11b^+^Ly6G^+^ cells. GR‐1 and Ly6G were used in separate panels and were not combined to define neutrophils.

### 2.9 Hydroxyproline Measurements of Lung Tissue

Hydroxyproline measurements were performed using reagents and protocols provided with the Hydroxyproline (HYP) Colourimetric Assay Kit (Elabscience, Texas, USA). Snap‐frozen inferior/postcaval lobes were weighed and added to a 2 mL U‐bottom Eppendorf with a 5 mm stainless steel bead. 10 µl of 6N HCl per mg of tissue was added to the 2 mL Eppendorf, and tissues were homogenised with a TissueLyser LT (Qiagen, Germany, EU) for 2–5 min at 25 Hz.

250 µL of lung homogenate (equivalent to 25 mg of tissue) was transferred to a 1.5 mL screw‐cap microtube and boiled in a fume hood at 95°C for 6 h. Tubes were then cooled in an ice‐water bath for 5 min, and 250 µL pH adjusting solution A plus 125 µL of pH‐adjusting solution B were added. After mixing evenly, samples were topped up to 2.5 mL with double‐distilled H_2_O and 250 µL of hydrolysate was transferred to a new 1.5 mL tube. The hydrolysate was then decolourised by adding 2.5 mg of clarificant, centrifuging at 1500×*g* for 10 min, and transferring the supernatant to a new 1.5 mL tube. The hydroxyproline stock was diluted in distilled H_2_O to a working concentration of 100 µg/mL, and a series of dilutions was performed in distilled H_2_O (10 µg/mL top standard) to generate a standard curve. 100 µL of sample or standard was transferred to a new 1.5 mL tube, and 50 µL of oxidant agent was added. Tubes were vortexed and incubated at room temperature (RT) for 15 min before adding 100 µL of chromogenic reagent. Samples were incubated in a fume hood at 60°C for 16 min, then cooled in an ice bath. 50 µL from each tube were transferred to a 96‐well plate for Enzyme‐Linked Immunosorbent Assays (ELISA), and optical density (OD) readings were taken at 558 nm on a CLARIOstar PLUS plate reader (BMG LABTECH, Germany, EU). Hydroxyproline concentrations were calculated in GraphPad Prism via nonlinear regression analysis. OD558 values were interpolated to the standard curve with a 99% confidence interval.

### 2.10 Histology of FFPE Lung Sections

Formalin‐fixed tissues stored in 70% ethanol initially underwent processing through an Epredia STP 120 Spin Tissue Processor (Fisher Scientific) by rotating tissues on a gradient of increasing concentrations of Ethanol (Fisher Scientific) to displace any residual water in tissues. Tissues were then moved in a xylene substitute (Sub‐X Clearing Medium, Leica, Germany, EU) to remove any ethanol residue before embedding in paraffin (Leica).

After processing, tissues were embedded in paraffin blocks via an Epredia Histostar embedding station (Fisher Scientific). Formalin‐fixed paraffin‐embedded (FFPE) tissues were sectioned into 5 µm‐thick sections using a Thermo Finesse ME+ rotary microtome (Thermo Fisher). Sections were mounted onto superfast slides (Thermo Fisher) and air‐dried overnight before dewaxing and staining.

### 2.11 Masson's Trichrome Staining

FFPE sections were dewaxed, rehydrated, and stained using a Masson's trichrome kit (Generon Clinisciences, UK). Collagen was visualised following standard staining procedures. Sections were scanned with a Zeiss AxioScan 7 slide scanner at 20× magnification (Zeiss, Germany, EU).

### 2.12 Ashcroft Scoring

In QuPath v0.6.0‐rc3 [33], scanned slides were loaded, and a 2300 × 2300 micron grid was overlaid onto the sections. Only 2300 × 2300 micron regions of interest (ROI) where tissues occupied 25% or more of the field of view were scored. Each section was scored as follows: 0: normal lung, 1–2: minimal fibrous thickening of alveolar or bronchiolar walls, 3–4: moderate thickening of walls without obvious damage to lung architecture, 5–6: increased fibrosis with definite damage to lung structure and formation of fibrous bands or small fibrous masses, 7: Severe distortion of structure and large fibrous areas (e.g. “Honeycomb lung”), 8: Total fibrous obliteration of the field. Scoring was performed by two individuals blinded to the study to eliminate bias. Each 2300 × 2300 micron ROI in the grid received a score, and all scores from one section were averaged to obtain the overall fibrosis score of the slide.

### 2.13 Halo Machine Learning

Using HALO v3.4 (Indica Labs, USA), trained MiniNet classifiers segmented tissue into fibrotic, healthy, airway, and vascular regions. Training parameters were set at 0.5 µm/px resolution with a minimum object size of 1000 µm^2,^ and 15,000 training iterations were performed. The entire tissue annotation from each section was analyzed with the trained classifier, and the size in µm^2^ of the relative ROIs was obtained. Percentages of fibrotic areas were then calculated relative to the total surface of the tissue minus the airway, blood vessel, and background regions.

### 2.14 Statistical Analysis

Data were statistically analysed in GraphPad Prism (v10.4.1) via one‐way ANOVA with Tukey's multiple comparison test. Data are reported as mean ± standard deviation (SD) with a statistical significance of: ns = nonsignificant, *p < 0.05, **p < 0.01, ***p < 0.001, ****p < 0.0001.

Unless otherwise stated, each data point represents an individual mouse. Experiments described as pooled from two independent repeats were performed twice using the same design and analyzed as individual mouse‐level data after pooling; the total *n* per group is shown in the relevant figure panels or legends.

## 3 Results

### 3.1 *Hpb* Infection or HES Administration Prevents BLM‐induced Fibrosis

To determine whether *Hpb* infection could prevent BLM‐induced lung fibrosis, we infected mice with *Hpb* 28 or 7 days before receiving BLM oropharyngeally. Mice were then culled 21 days after BLM for fibrotic measurements (Figure 1A). To assess fibrosis, lung tissue was analysed for collagen content via Masson's Trichrome staining. Manual Ashcroft scoring and machine learning analysis both showed a clear induction of fibrotic pathology with BLM treatment, which was reduced on *Hpb* infection, whether the infection occurred 7 or 28 days before BLM administration (Figure 1B–E).

**FIGURE 1:**
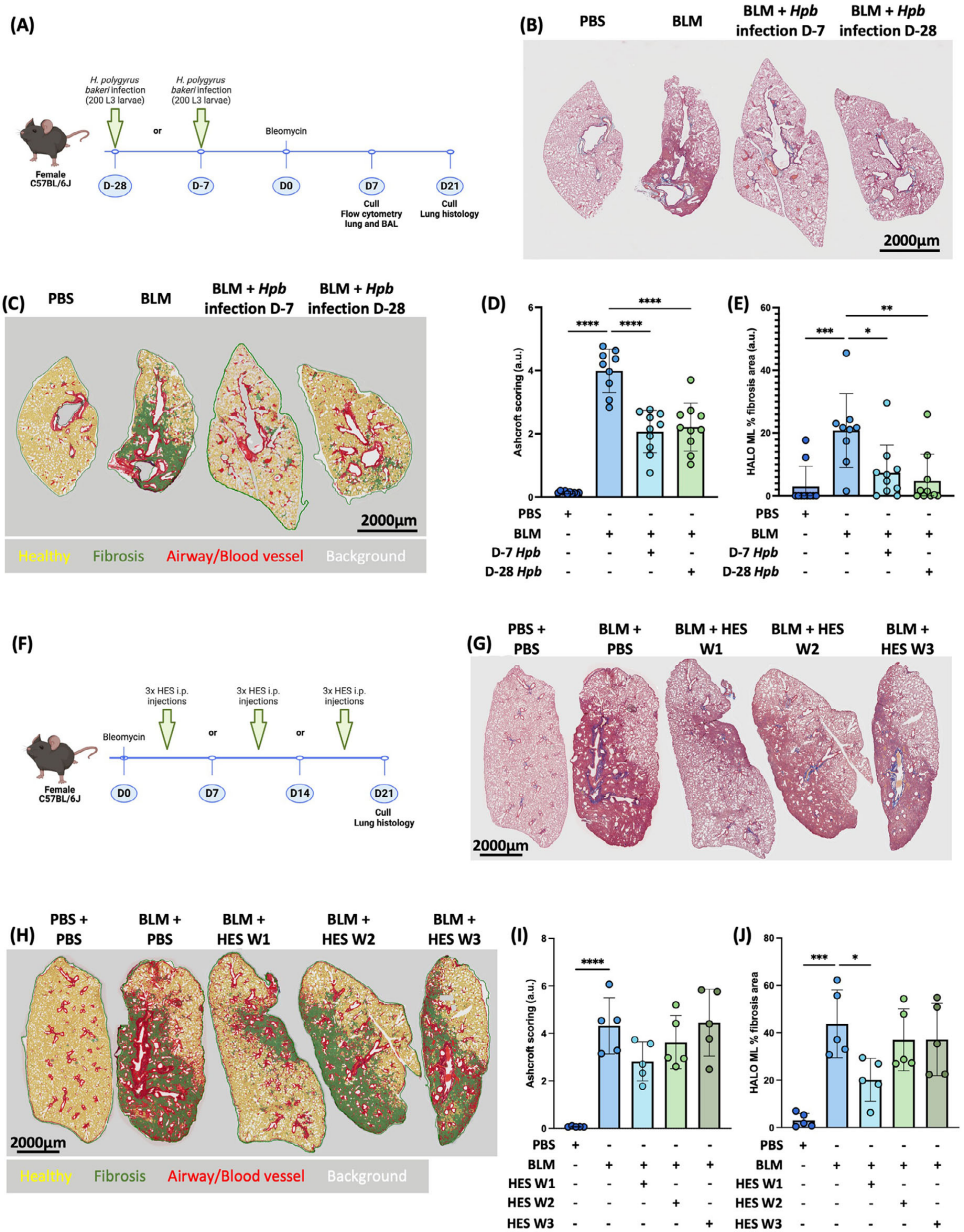
*Hpb* and its HES secretions prevent BLM‐induced fibrosis in the lung. (A) Mice were infected with *Hpb* larvae 7 (D‐7) or 28 (D‐28) days before the oropharyngeal administration of 1.5 mg/kg BLM. (B) Representative Masson's Trichrome‐stained lung sections. (C) Slides were analysed by AI‐based tissue segmentation in HALO using a MiniNet classifier to quantify fibrosis; representative overlays show healthy tissue (yellow), fibrosis (green), and airways/vessels (red). (D) Blinded Ashcroft scoring. (E) Fibrotic tissue percentages from HALO analysis. (F) C57BL/6J mice received 1.5 mg/kg BLM plus three intraperitoneal injections of 10 µg HES (or vehicle) during weeks 1–3; lungs were analysed 21 days post‐BLM. (G) Representative Masson's Trichrome sections. (H) HALO MiniNet analysis and representative overlays. (I) Blinded Ashcroft scoring. (J) Fibrotic tissue percentages from HALO analysis. For *Hpb* infection, data are pooled from two independent repeats (each with group sizes of *n* = 4–5) to give total group sizes of *n* = 9–10. For HES treatment, data were derived from a single experiment with a group size of *n* = 5. Data are represented as mean ± SD. Statistical analysis was performed via one‐way ANOVA with Tukey's multiple comparison test. *p < 0.05, **p < 0.01, ***p < 0.001, ****p < 0.0001.

To further determine if the preventative anti‐fibrotic effect of *Hpb* was due to its excretory/secretory products (HES), mice received BLM oropharyngeally and were treated with HES intraperitoneally. To determine the timing of the effect, HES was administered during the first, second, or third week post‐BLM (Figure 1F).

At day 21‐post BLM, quantitative analysis of Masson's Trichrome‐stained slides via blinded Ashcroft scoring and machine learning showed that HES administration in week 1 reduced fibrosis, although this difference reached significance only in machine learning quantitation, while only a nonsignificant trend in the same direction was seen in the Ashcroft scoring (Figure 1G–J). In contrast, HES treatment during weeks 2 and 3 of the model did not show any change when compared with the BLM‐positive controls.

These data establish the first step of the study: *Hpb* infection protects against BLM‐induced fibrosis, and soluble HES products are sufficient to reproduce this protection when administered during the early inflammatory phase. The loss of protection when HES was administered in later phases suggested that helminth‐derived factors prevent the initiation of fibrosis rather than reversing lung scarring.

### 3.2 Dual IL‐33 Blockade via HpARI2 and HpBARI_Hom2 Prevents, but Does Not Reverse, Lung Fibrosis

Having shown that HES was sufficient to prevent fibrosis, we next asked whether defined HES‐derived IL‐33 inhibitors HpARI2 and HpBARI_Hom2 could account for this effect. Recombinant HpARI2 and HpBARI_Hom2 proteins were administered to mice during the first week of the BLM‐induced fibrosis model (Figure 2A). At day 21 post‐BLM, both Ashcroft scoring and machine learning‐based analysis confirmed that HpARI2 and HpBARI_Hom2 treatment during the first week post‐BLM attenuated fibrotic pathology (Figure 2B–E). Furthermore, hydroxyproline levels (as a measure of collagen deposition) were significantly lower in treated animals, supporting the histological findings (Figure 2F). These results demonstrate that early suppression of IL‐33 signalling via HpARI2 and HpBARI_Hom2 can effectively prevent the development of pulmonary fibrosis in the BLM model. This result also shows that the antifibrotic activity of HES can be reproduced by a defined parasite‐derived protein pair that targets IL‐33 signalling.

**FIGURE 2:**
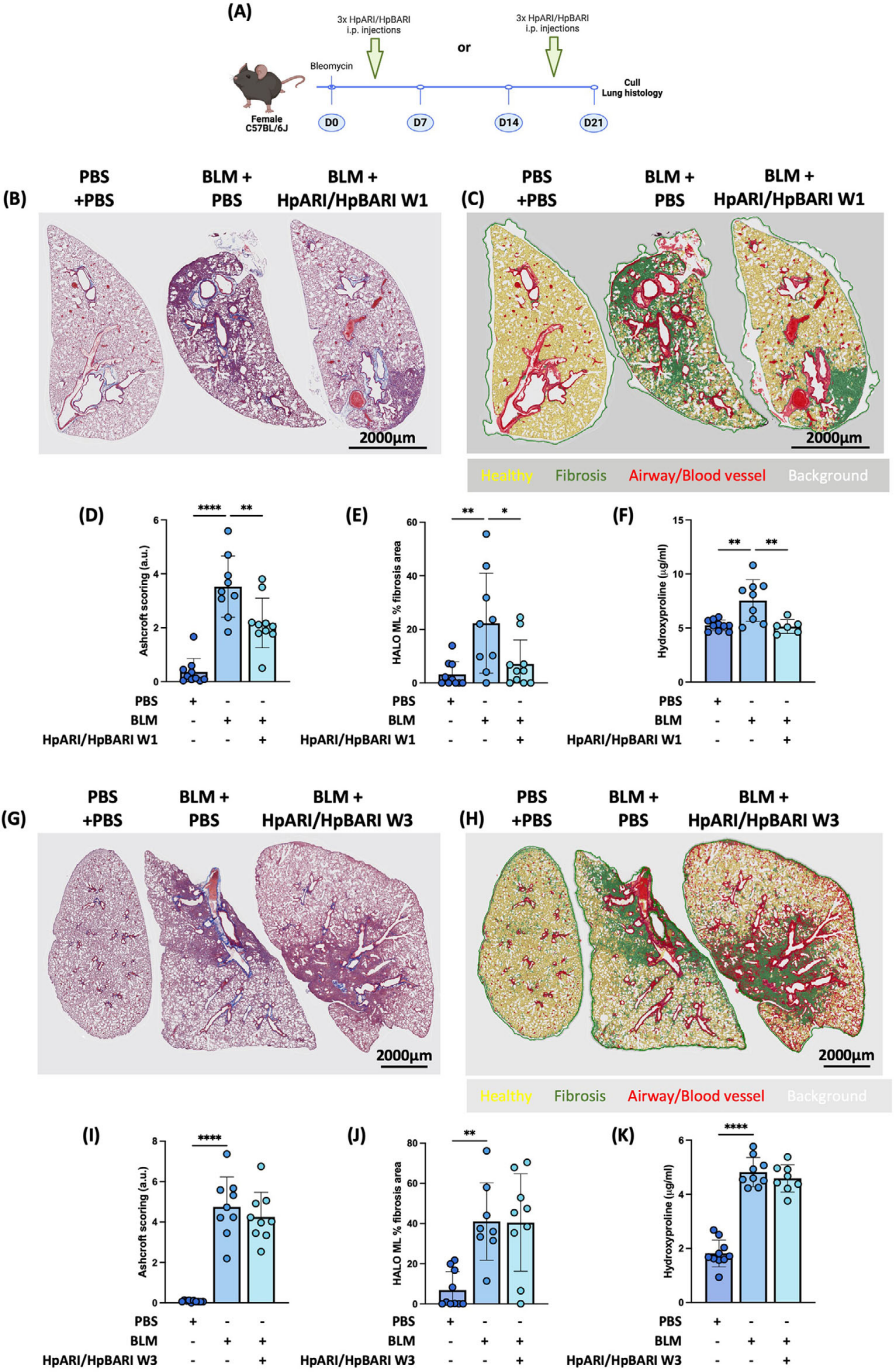
HpARI2 and HpBARI_Hom2 prevent BLM‐induced lung fibrosis. (A) C57BL/6J mice were treated with 1 oropharyngeal challenge of 1.5 mg/kg BLM under light isoflurane anaesthesia. HpARI2 and HpBARI_Hom2 (10 µg of each protein), or vehicle control, were co‐injected intraperitoneally on days 0, 2, and 4 (week 1 (W1) inflammatory period) or days 14, 16, and 18 (week 3 (W3) pro‐fibrotic period) post‐BLM. (B, G) Representative Masson's Trichrome‐stained lung sections. (C, H) Representative images with overlay from HALO machine learning analysis, whereby yellow is healthy tissue, green is fibrosis, and red is airways and blood vessels. (D, I) Blinded Ashcroft scoring of scanned Masson's Trichrome‐stained sections. (E, J) Percentages of fibrotic tissue calculated via HALO machine learning analysis. (F, K) Hydroxyproline measurements on total lung homogenates. For both administration time courses, data are pooled from two independent repeats (each with group sizes of *n* = 3–5) to give total group sizes of *n* = 6–10. Data are represented as mean ± SD. Statistical analysis was performed via one‐way ANOVA with Tukey's multiple comparison test. *p < 0.05, **p < 0.01, ***p < 0.001, ****p < 0.0001.

We then tested whether the same dual blockade could reverse established disease by administering the same recombinant proteins after the early inflammatory phase during later stages of the disease (days 7, 9 and 11 or 14, 16, and 18 post‐BLM) (Figure S1A; Figure 2A). In contrast to week 1 treatment, later administration failed to reduce fibrosis as assessed by Ashcroft scoring, machine learning, and hydroxyproline content (Figure S1B–E; Figure 2G–K).

Thus, HpARI2+HpBARI_Hom2 reveals a timing‐dependent role for IL‐33 in fibrotic development: blocking the IL‐33 pathway prevents BLM‐induced fibrosis during disease initiation but is insufficient once fibrotic pathology has been established.

### 3.3 Early HpARI2+HpBARI_Hom2 Administration Suppresses Immune Responses to BLM

To determine how early dual IL‐33 blockade altered the inflammatory phase of the model, mice were treated with BLM on day 0 and HpARI2 + HpBARI_Hom2 on days 0, 2, and 4, and lung tissues were harvested for flow cytometric analysis of immune cell subsets 7 days post‐BLM. Lung flow‐cytometry analyses at day 7 are shown in Figures S2 and S3. Early HpARI2+HpBARI_Hom2 treatment reduced BLM‐induced lung cellular infiltration (Figure S3B). Total lung cell and lung CD4^+^Lineage^+^CD45^+^ T cell numbers were significantly increased by BLM (Figure S3B–G), while HpARI2+HpBARI_Hom2 co‐treatment resulted in these numbers staying at baseline. HpARI2+HpBARI_Hom2 treatment also significantly reduced ICOS^+^CD25^+^CD4^−^Lineage^−^ ILC2 numbers and their ST2 mean fluorescence intensity as opposed to BLM‐treated mice (Figure S3H–J). The masking of ST2 by HpBARI_Hom2 binding, preventing detection of ST2, has been previously reported [29, 34], and indicates that HpBARI_Hom2 can act in the lung after intraperitoneal administration. ILC2 activation often results in IL‐5 and IL‐13 production; however, neither of these cytokines reached the limit of detection in BALF samples in these experiments. ILC2 activation can also be measured by cell size, as assessed by FSC‐A measurements by flow cytometry [34]. Here, BLM increased ILC2 size compared with PBS controls, whereas in the HpARI2+HpBARI_Hom2 treatment group, ILC2 size did not differ from baseline (Figure S3K).

To further assess the immune response after BLM treatment and HpARI2+HpBARI_Hom2 administration, we collected BALF cells at days 7 or 21 post‐BLM administration. In the BALF, BLM exposure significantly increased total cell numbers at both week 1 and week 3 post‐administration, while early HpARI2+HpBARI_Hom2 treatment reduced this effect (Figure 3A). At week 1, BLM significantly increased BALF and lung eosinophil numbers, an effect which was blunted by HpARI2+HpBARI_Hom2 administration (Figure 3B–D; Figures S4A–C and S5). BALF alveolar macrophages showed decreased frequency but increased total numbers following BLM, with a progressive rise in number between weeks 1 and 3, while HpARI2+HpBARI_Hom2 treatment significantly reduced macrophage numbers at both time points (Figure 3B,E,F). BALF (but not lung) neutrophil frequencies and numbers were also increased post‐BLM, while HpARI2+HpBARI_Hom2 treatment slightly reduced this effect (Figure 3G–I; Figures S4D–F and S5).

**FIGURE 3:**
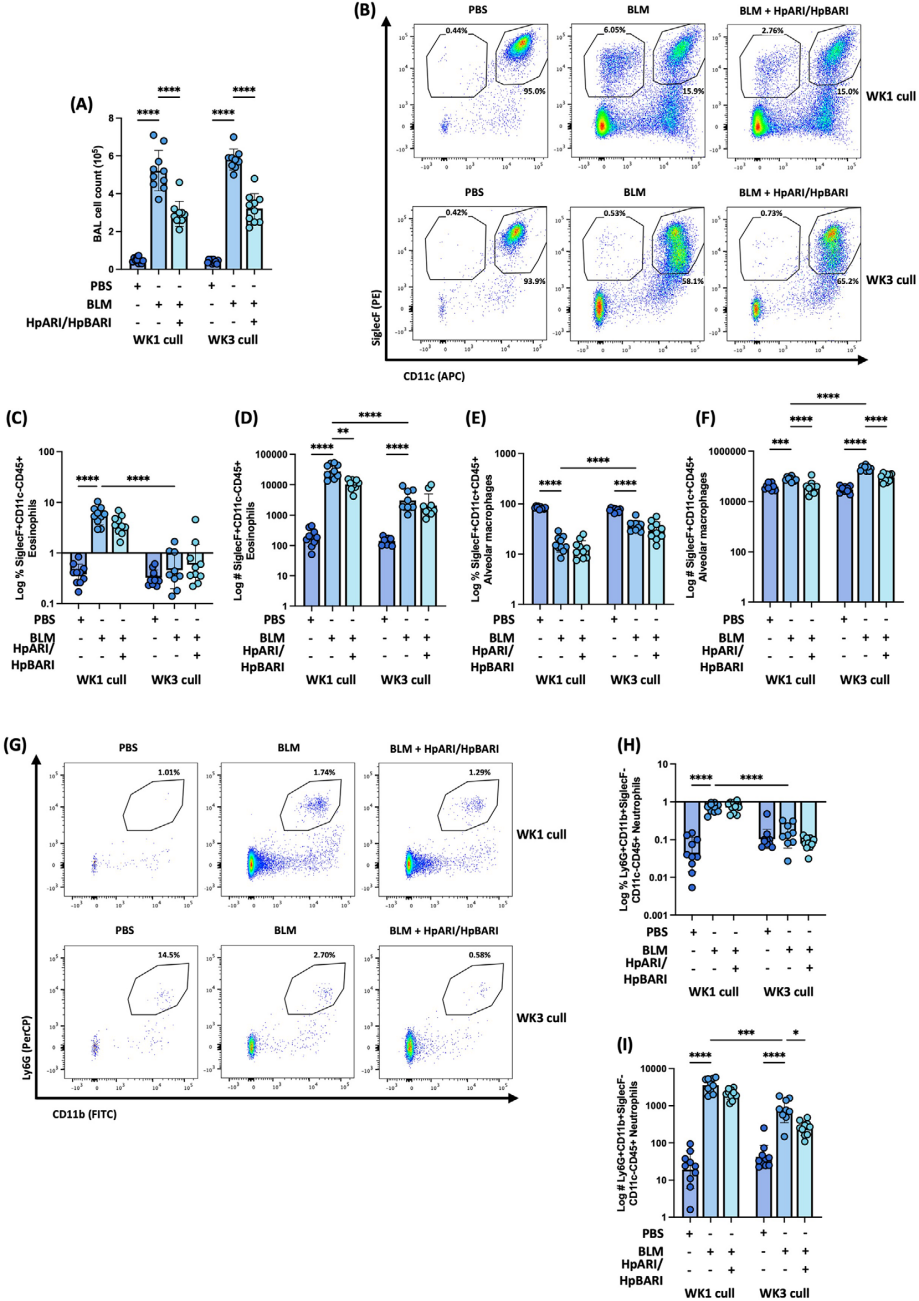
HpARI2 and HpBARI_Hom2 reduce BLM‐induced inflammation in the BAL at 7 (WK1) and 21 (WK3) days post‐BLM treatment. C57BL/6J mice were treated with 1.5 mg/kg oropharyngeal BLM, while a mix of HpARI2 and HpBARI_Hom2 (10 µg each), or vehicle control, was injected intraperitoneally on days 0, 2, and 4. BAL of mice was analysed 7 or 21 days post‐BLM administration. (A) BAL total cell counts. (B) Representative flow cytometry plots of CD11c versus SiglecF, on gated live CD45^+^ cells, showing BAL SiglecF^+^CD11c^−^CD45^+^ eosinophils and SiglecF^+^CD11c^+^CD45^+^ alveolar macrophages. Percentages (C) and numbers (D) of SiglecF^+^CD11c^−^CD45^+^ eosinophils. Percentages (E) and numbers (F) of SiglecF^+^CD11c^+^CD45^+^ alveolar macrophages. (G) Representative flow cytometry plots of CD11b versus Ly6G, on gated SiglecF^−^CD11c^−^CD45^+^ cells, showing BAL Ly6G^+^CD11b^+^SiglecF^−^CD11c^−^CD45^+^ neutrophils. Percentages (H) and numbers (I) of Ly6G^+^CD11b^+^SiglecF^−^CD11c^−^CD45^+^ neutrophils. At each time point, data are pooled from two independent repeats (each with group sizes of *n* = 4–5) to give total group sizes of *n* = 9–10. Data are represented as mean ± SD. Statistical analysis was performed via one‐way ANOVA with Tukey's multiple comparison test. *p < 0.05, **p < 0.01, ***p < 0.001, ****p < 0.0001.

Together, these immune data indicate that early HpARI2+HpBARI_Hom2 treatment dampens a broad inflammatory programme involving ILC2s, eosinophils, macrophages and neutrophils during the period in which fibrosis is initiated.

### 3.4 IL‐33trap Confirms Timing‐Dependent Prevention, but Not Reversal of BLM‐Induced Lung Fibrosis

To confirm that the anti‐fibrotic effects observed with HpARI2+HpBARI_Hom2 were due to IL‐33 inhibition rather than off‐target effects of recombinant proteins, we employed a recombinant IL‐33trap, which has also proven effective in blocking IL‐33 in vivo [30]. To assess if other epithelial cytokines are involved, we administered the TSLP‐blocking reagent TSLPtrap [31].

Consistent with the HpARI2+HpBARI_Hom2 data, mice received oropharyngeal BLM and IL‐33trap in either the first or third weeks post‐BLM (Figure 4A). Quantitative assessment using Ashcroft scoring showed that IL‐33trap during week 1 significantly reduced fibrosis relative to BLM controls, while it did not quite reach significance in machine learning analysis (*p* = 0.07) (Figure 4B–E). Conversely, IL‐33trap administration in week 3 did not affect fibrosis measurements (Figure 4B–E).

**FIGURE 4:**
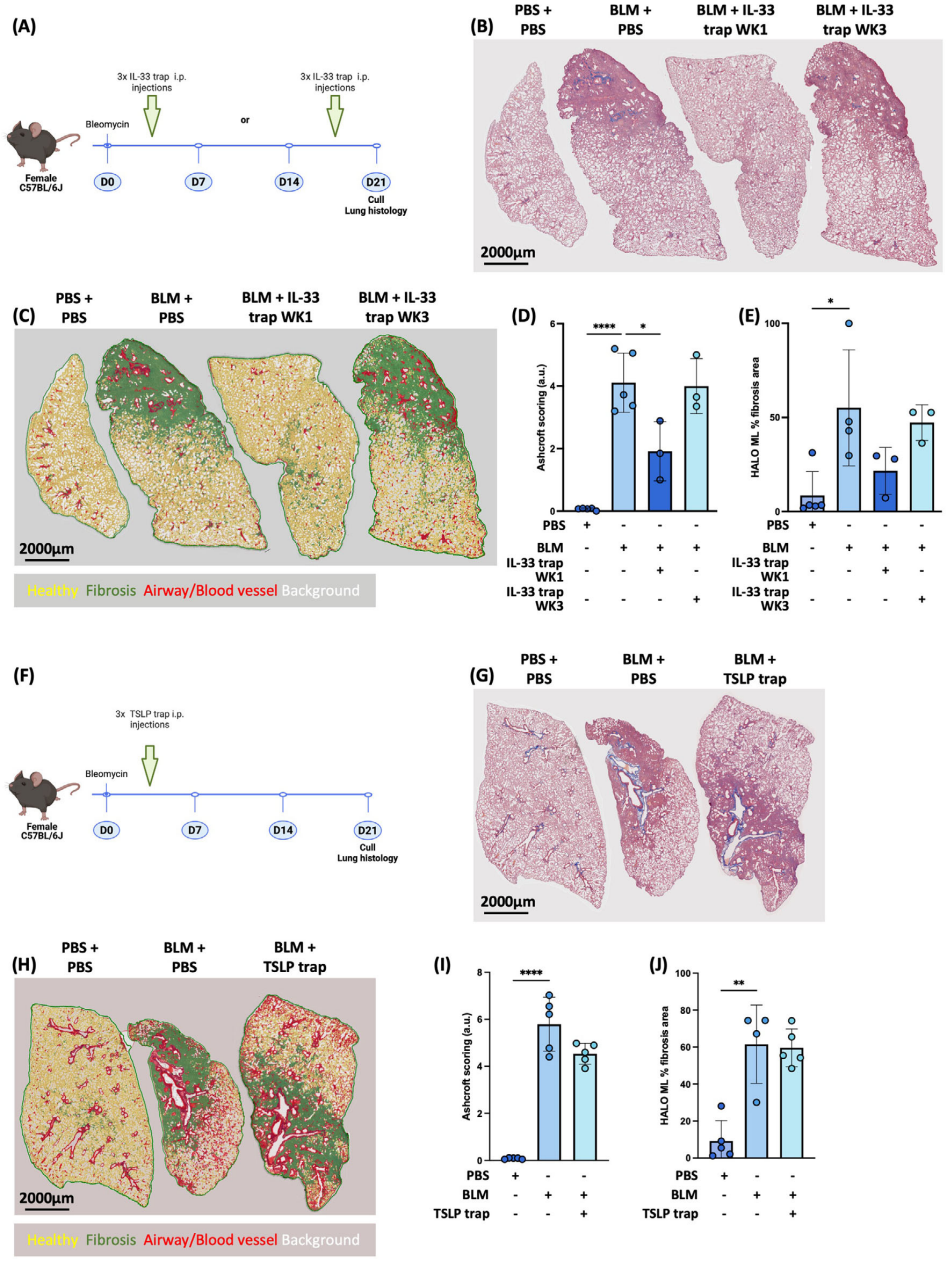
IL‐33trap, but not TSLPtrap, can prevent BLM‐induced lung fibrosis. (A) C57BL/6J mice were treated with 1 oropharyngeal challenge of 1.5 mg/kg BLM under light isoflurane anaesthesia. IL‐33trap (20 µg), or vehicle control, was intraperitoneally injected on days 0, 2, and 4. Alternatively, IL‐33 trap was administered on days 14, 16, and 18 post‐BLM. (B) Representative Masson's Trichrome‐stained lung sections. (C) HALO MiniNet AI analysis showing healthy tissue (yellow), fibrosis (green), and airways/vessels (red). (D) Blinded Ashcroft scoring. (E) Fibrotic tissue percentages from HALO analysis. (F) Mice received BLM followed by TSLPtrap (50 µg) or vehicle on days 0, 2, and 4 post‐BLM. (G) Representative Masson's Trichrome sections. (H) HALO MiniNet analysis and representative overlays as in (C). (I) Blinded Ashcroft scoring. (J) Fibrotic tissue percentages from HALO analysis. For each treatment course, data are from single experiments with a group size of *n* = 3–5. Data are represented as mean ± SD. Statistical analysis was performed via one‐way ANOVA with Tukey's multiple comparison test. *p < 0.05, **p < 0.01, ***p < 0.001, ****p < 0.0001.

TSLPtrap administration in the first week post‐BLM did not affect fibrosis measurements, confirming that these effects are dependent on blocking IL‐33 (Figure 4F–J).

These findings confirm that IL‐33 blockade is protective when applied during inflammatory initiation, but that neutralising IL‐33 after fibrosis is established is not sufficient to reverse fibrotic lung pathology.

## 4 Discussion

Our study establishes in the BLM model that the anti‑fibrotic benefits of *Hpb* infection are due to its secretions, which block IL‑33 binding to its receptor ST2 via two key proteins, HpARI2 and HpBARI_Hom2. When administered during the early inflammatory phase, IL‐33 blockade robustly prevents lung fibrosis, while reducing ILC2, eosinophil, macrophage and neutrophil accumulation. However, neither whole HES nor IL‑33 blockade is effective once fibrosis is established, demonstrating that IL‑33 is essential for initiating, but not maintaining fibrotic pathology. The lack of protection by TSLPtrap further confirms that these anti‐fibrotic effects are specific to IL‐33 neutralisation rather than off‐target effects of recombinant protein administration. Here, we also demonstrated that although inflammation in the BALF is resolving at week 3 post‐BLM, it is still present, and blocking IL‐33 early in the model can negatively regulate it.

The central novelty of this study is therefore not simply that IL‐33 contributes to BLM‐induced fibrosis, but that a parasite infection can alter lung fibrotic pathology through defined secreted molecules. By moving from live *Hpb* infection to HES and then to recombinant HpARI2+HpBARI_Hom2, we separate the protective effect from nonspecific infection‐associated immunomodulation and identify a defined parasite‐derived molecular strategy that reproduces this activity. Importantly, this strategy blocks both sides of the IL‐33 signalling axis: HpARI2 binds IL‐33 directly, while HpBARI_Hom2 binds ST2. Furthermore, by using the IL‐33trap protein, we could replicate these effects and confirm that the reduction of fibrosis was due to IL‐33 inhibition.

BLM was chosen to model IPF due to its reproducibility and rapid induction of lung fibrosis, despite its self‐limiting nature that contrasts with human progressive fibrosis [35, 36]. BLM, an anti‐tumour glycopeptide from *Streptomyces verticillus*, causes fibrosis as a toxic side effect [37, 38, 39, 40, 41, 42]. Consistent with prior studies, BLM exposure induced cellular infiltration, collagen deposition, and lung architectural loss, confirming the suitability of the model to test the anti‐fibrotic effects of *Hpb* and its secretions [43, 44, 45].

As hypothesised, *Hpb* infection reduced BLM‐induced fibrosis, suggesting systemic immunomodulation via secreted proteins or an anti‐parasitic Th2 response modulating lung inflammation. This anti‐fibrotic effect parallels findings in co‐infection models with *Schistosoma mansoni*, where *Hpb* reduced fibrotic granulomas without affecting parasite burden [22], and recent work showing that *Hpb* prevents BLM‐induced subcutaneous fibrosis by modulating collagen secretion [23]. These data support the idea that *Hpb* evolved mechanisms to evade host immunity through fibrosis modulation.

We assessed if the anti‐fibrotic effects of *Hpb* were due to its secretions (HES) and showed that HES reproduces the effect of infection in the lung. Therefore, our data argue that soluble parasite‐derived factors, rather than parasite burden or nonspecific infection‐associated changes alone, are sufficient to alter the fibrotic outcome.

The HES timing experiment further indicates that these factors act during inflammatory initiation. HES treatment in week 1 prevented fibrosis, whereas treatment during weeks 2 or 3 did not. This supports a model in which parasite‐derived products reduce the upstream inflammatory signals that license fibrogenesis, rather than directly degrading collagen or reversing established tissue remodelling. Since *Hpb* mainly resides in the gastrointestinal tract, where it induces barrier damage and the release of IL‐33 [46, 47], it likely evolved immunomodulatory proteins targeting these cytokines rather than collagen itself.

These findings are consistent with previous work showing reduced fibrosis after utilising genetic ablation of the IL‐33 receptor ST2, administration of anti‐IL‐33 antibodies, or lentiviral overexpression of the sST2 decoy receptor during the early inflammatory phase of the response to BLM [11, 15, 16, 17, 18]. While IL‐33 inhibition is clearly effective in preventing the initiation of fibrosis, our results also show that these treatments could not reverse established fibrosis when given in the late phase of the model [16]. Because late HpARI2+HpBARI_Hom2 or IL‐33trap treatment did not reduce established collagen deposition, the reduced fibrosis seen after early treatment most likely reflects suppression of upstream inflammatory pathways rather than degradation of extracellular matrix or direct reversal of fibroblast activation. Given that IL‐33 blockade reduced multiple inflammatory populations, we cannot exclude that the anti‐fibrotic phenotype is secondary to broad suppression of early lung inflammation rather than a direct action on extracellular matrix‐producing cells.

Early IL‐33 blockade also reversed the BLM‐induced increase in lung eosinophils and BALF neutrophils, eosinophils, and alveolar macrophages. Similarly, previous studies have shown that mice deficient in ST2 had reduced neutrophil and macrophage responses to BLM, while IL‐33 administration or transgenic expression increased BALF macrophage, neutrophil and/or eosinophil numbers [11, 17, 18]. These data support our hypothesis that IL‐33 has a central role during pro‐fibrotic early lung inflammation and that blocking its signalling can prevent resulting lung fibrosis.

Additionally, TSLP inhibition using TSLPtrap administration was also evaluated in the BLM model; however, this did not affect fibrotic pathology. One study using TSLPR‐deficient mice or anti‐TSLP antibodies showed no changes in fibrosis scores but reported increased airway inflammation and morbidity [48], while a second study reported contrasting results, showing reduced fibrosis in TSLPR‐deficient mice during the fibrotic phase [49]. These discrepancies may stem from methodological differences, particularly in the route of BLM administration (oropharyngeal vs. intratracheal), which can influence BLM potency and downstream cytokine levels [44]. Moreover, additional studies examining the in vivo half‐life of TSLPtrap and its efficacy at higher concentrations are required to determine whether the observed lack of effect reflects insufficient TSLP blockade or a limited role for TSLP in BLM‐induced lung fibrosis. Therefore, future studies should compare these routes and assess TSLP blockade to clarify its role in fibrosis development.

From a translational perspective, these findings support IL‐33/ST2 blockade as a timing‐dependent intervention rather than as a late‐stage anti‐fibrotic treatment. Patients with established IPF generally present after matrix deposition and architectural remodelling are already present, and our late‐treatment data argue that IL‐33 neutralisation alone would be unlikely to reverse such disease. The most plausible clinical setting would therefore be early or preventative intervention in individuals with active epithelial injury, elevated IL‐33/ST2 pathway biomarkers, or high risk of progressive fibrosis. The parasite‐derived nature of HpARI2 and HpBARI_Hom2 also broadens the translational value of the work: it demonstrates that host–pathogen co‐evolution can reveal molecular routes to dampen pro‐fibrotic alarmin signalling.

In conclusion, our study shows that infection with Hpb can protect the lungs from BLM‐induced lung fibrosis by selectively targeting IL‐33 via HpARI2 and HpBARI_Hom2. However, once fibrosis has developed, blocking IL‐33 cannot reverse this process, highlighting that IL‐33 is important at the start of the disease, but not later. These results suggest that early treatment targeting IL‐33 could be a promising way to prevent lung fibrosis before it becomes irreversible in patients with early symptoms of IPF.

### 4.1 Data Limitations and Perspectives

Several reviewer‐requested experiments were not performed because they would require extensive additional in vivo studies which were outside the scope of this project. Specifically, we did not repeat experiments solely to resolve differences between Ashcroft and machine‐learning quantification, nor did we perform further flow cytometry staining for additional inflammatory markers (such as IL‐5, IL‐13, GATA3, CD64 or expanded macrophage/neutrophil markers). Therefore, future studies could focus on the inflammatory phenotype of these immune cell populations during BLM fibrosis and the effect of suppression by IL‐33 blockade.

Lung qPCR and BALF ELISA for fibrosis‐associated markers were also carried out but were not informative in this study. At day 21, BALF IL‐5 and IL‐13 were below detection, and qPCR analysis of fibrosis‐associated transcripts such as Col1a1 and Acta2/alpha‐SMA did not differ between baseline and the BLM group. Because day 21 captures the established or resolving phase of this self‐limiting model rather than the early alarmin‐driven inflammatory phase, these measurements were inconclusive. Future studies could focus on the effects of IL‐33 inhibition in the inflammatory phase of the BLM model.

Finally, the BLM model is an acute, injury‐driven and partially self‐resolving model, whereas human IPF is chronic and usually diagnosed after fibrotic remodelling is established. The present data therefore support a preventative or early‐intervention concept for IL‐33 pathway blockade, not reversal of established IPF. Testing parasite‐derived IL‐33 inhibitors in additional progressive fibrosis models, and in combination with approved anti‐fibrotic drugs would help define whether this mechanism has translational potential beyond the early inflammatory phase of BLM‐induced injury.

## Author Contributions

Investigations: S.D.C., T.F., S.H.H., D.J.S., T.B., I.N., S.K., A.R.S., and H.J.M. Supervision: E.S.C., J.S.C.A., and H.J.M. Funding acquisition: E.S.C., H.J.M. Writing – original draft: S.D.C. Writing – review and editing: E.S.C., J.S.C.A., and H.J.M.

## Funding

This work was supported by Medical Research Scotland and AstraZeneca Ltd PhD studentship [PHD‐50198‐2020].

## Conflicts of Interest

The authors declare no conflicts of interest.

## Supporting information




**Supporting File**: eji70291‐sup‐0001‐SuppMat.docx.

## Data Availability

The data that support the findings of this study are available from the corresponding author upon reasonable request.

## References

[1] C. Upagupta , C. Shimbori , R. Alsilmi , and M. Kolb , “Matrix Abnormalities in Pulmonary Fibrosis,” European Respiratory Review 27, no. 148 (2018): 180033, 10.1183/16000617.0033-2018. PMC9489108 29950306

[2] P. D. Blanc , I. Annesi‐Maesano , J. R. Balmes , et al., “The Occupational Burden of Nonmalignant Respiratory Diseases. An Official American Thoracic Society and European Respiratory Society Statement,” American Journal of Respiratory and Critical Care Medicine 199, no. 11 (2019): 1312–1334, 10.1164/rccm.201904-0717ST. PMC6543721 31149852

[3] R. Hubbard , S. Lewis , K. Richards , I. Johnston , and J. Britton , “Occupational Exposure to Metal or Wood Dust and Aetiology of Cryptogenic Fibrosing Alveolitis,” The Lancet 347, no. 8997 (1996): 284–289, 10.1016/S0140-6736(96)90465-1. 8569361

[4] R. Hubbard , M. Cooper , M. Antoniak , et al., “Risk of Cryptogenic Fibrosing Alveolitis in Metal Workers,” The Lancet 355, no. 9202 (2000): 466–467, 10.1016/S0140-6736(00)82017-6. 10841131

[5] W. Bae , C. H. Lee , J. Lee , Y. W. Kim , K. Han , and S. M. Choi , “Impact of Smoking on the Development of Idiopathic Pulmonary Fibrosis: Results From a Nationwide Population‐based Cohort Study,” Thorax 77, no. 5 (2022): 470–476, 10.1136/thoraxjnl-2020-215386. 34593614

[6] X. Xu , J. Zhang , and H. Dai , “IL‐25/IL‐33/TSLP Contributes to Idiopathic Pulmonary Fibrosis: Do Alveolar Epithelial Cells and (myo)Fibroblasts Matter?,” Experimental Biology and Medicine 245, no. 10 (2020): 897–901, 10.1177/1535370220915428. PMC7268928 32249602

[7] C. S. Hardman , V. Panova , and A. N. McKenzie , “IL‐33 Citrine Reporter Mice Reveal the Temporal and Spatial Expression of IL‐33 During Allergic Lung Inflammation,” European Journal of Immunology 43, no. 2 (2013): 488–498, 10.1002/eji.201242863. PMC3734634 23169007

[8] F. Y. Liew , J. P. Girard , and H. R. Turnquist , “Interleukin‐33 in Health and Disease,” Nature Reviews Immunology 16, no. 11 (2016): 676–689, 10.1038/nri.2016.95. 27640624

[9] M. Abdo , F. Pedersen , A. M. Kirsten , et al., “Association of Airway Inflammation and Smoking Status With IL‐33 Level in Sputum of Patients With Asthma or COPD,” European Respiratory Journal 64, no. 3 (2024): 2400347, 10.1183/13993003.00347-2024. PMC11424925 39147409

[10] J.‐U. Lee , H. S. Chang , H. J. Lee , et al., “Upregulation of Interleukin‐33 and Thymic Stromal Lymphopoietin Levels in the Lungs of Idiopathic Pulmonary Fibrosis,” BMC Pulmonary Medicine 17, no. 1 (2017): 39, 10.1186/s12890-017-0380-z. PMC5312598 28202030

[11] D. Li , R. Guabiraba , A. G. Besnard , et al., “IL‐33 Promotes ST2‐dependent Lung Fibrosis by the Induction of Alternatively Activated Macrophages and Innate Lymphoid Cells in Mice,” Journal of Allergy and Clinical Immunology 134, no. 6 (2014): 1422–1432.e11, 10.1016/j.jaci.2014.05.011. PMC4258609 24985397

[12] I. G. Luzina , P. Kopach , V. Lockatell , et al., “Interleukin‐33 Potentiates Bleomycin‐induced Lung Injury,” American Journal of Respiratory Cell and Molecular Biology 49, no. 6 (2013): 999–1008, 10.1165/rcmb.2013-0093OC. PMC3931117 23837438

[13] O. S. Kotsiou , K. I. Gourgoulianis , and S. G. Zarogiannis , “IL‐33/ST2 Axis in Organ Fibrosis,” Frontiers in Immunology 9 (2018): 2432, 10.3389/fimmu.2018.02432. PMC6207585 30405626

[14] K. Oboki , T. Ohno , N. Kajiwara , H. Saito , and S. Nakae , “IL‐33 and IL‐33 Receptors in Host Defense and Diseases,” Allergology International 59, no. 2 (2010): 143–160, 10.2332/allergolint.10-RAI-0186. 20414050

[15] Q. Gao , Y. Li , X. Pan , X. Yuan , X. Peng , and M. Li , “Lentivirus Expressing Soluble ST2 Alleviates Bleomycin‐Induced Pulmonary Fibrosis in Mice,” International Immunopharmacology 30 (2016): 188–193, 10.1016/j.intimp.2015.11.015. 26604088

[16] K. E. Stephenson , J. Porte , A. Kelly , et al., “The IL‐33:ST2 Axis Is Unlikely to Play a central Fibrogenic Role in Idiopathic Pulmonary Fibrosis,” Respiratory Research 24, no. 1 (2023): 89, 10.1186/s12931-023-02334-4. PMC10035257 36949463

[17] M. Fanny , M. Nascimento , L. Baron , et al., “The IL‐33 Receptor ST2 Regulates Pulmonary Inflammation and Fibrosis to Bleomycin,” Frontiers in Immunology 9 (2018): 1476, 10.3389/fimmu.2018.01476. PMC6026799 29988569

[18] I. G. Luzina , E. M. Pickering , P. Kopach , et al., “Full‐length IL‐33 Promotes Inflammation but Not Th2 Response in Vivo in an ST2‐Independent Fashion,” Journal of Immunology 189, no. 1 (2012): 403–410, 10.4049/jimmunol.1200259. PMC3381874 22634619

[19] L. Richeldi , M. Kolb , S. Jouneau , et al., “Efficacy and Safety of nintedanib in Patients With Advanced Idiopathic Pulmonary Fibrosis,” BMC Pulmonary Medicine 20, no. 1 (2020): 3, 10.1186/s12890-019-1030-4. PMC6951000 31914963

[20] U. Costabel , C. Albera , L. H. Lancaster , et al., “An Open‐Label Study of the Long‐Term Safety of Pirfenidone in Patients With Idiopathic Pulmonary Fibrosis (RECAP),” Respiration 94, no. 5 (2017): 408–415, 10.1159/000479976. 28898890

[21] R. M. Maizels , J. P. Hewitson , J. Murray , et al., “Immune Modulation and Modulators in *Heligmosomoides polygyrus* Infection,” Experimental Parasitology 132, no. 1 (2012): 76–89, 10.1016/j.exppara.2011.08.011. PMC6485391 21875581

[22] L. E. Bazzone , P. M. Smith , L. I. Rutitzky , et al., “Coinfection With the Intestinal Nematode *Heligmosomoides polygyrus* Markedly Reduces Hepatic Egg‐induced Immunopathology and Proinflammatory Cytokines in Mouse Models of Severe Schistosomiasis,” Infection and Immunity 76, no. 11 (2008): 5164–5172, 10.1128/IAI.00673-08. PMC2573333 18710859

[23] A. Sekiguchi , C. Shimokawa , T. Kato , et al., “Inhibition of Skin Fibrosis via Regulation of Th17/Treg Imbalance in Systemic Sclerosis,” Scientific Reports 15, no. 1 (2025): 1423, 10.1038/s41598-025-85895-2. PMC11717915 39789188

[24] H. J. McSorley , N. F. Blair , K. A. Smith , A. N. McKenzie , and R. M. Maizels , “Blockade of IL‐33 Release and Suppression of Type 2 Innate Lymphoid Cell Responses by Helminth Secreted Products in Airway Allergy,” Mucosal Immunology 7, no. 5 (2014): 1068–1078, 10.1038/mi.2013.123. PMC4016792 24496315

[25] H. J. McSorley , M. T. O'Gorman , N. Blair , T. E. Sutherland , K. J. Filbey , and R. M. Maizels , “Suppression of Type 2 Immunity and Allergic Airway Inflammation by Secreted Products of the Helminth *Heligmosomoides polygyrus* ,” European Journal of Immunology 42, no. 10 (2012): 2667–2682, 10.1002/eji.201142161. PMC4916998 22706967

[26] J. P. Hewitson , A. C. Ivens , Y. Harcus , et al., “Secretion of Protective Antigens by Tissue‐Stage Nematode Larvae Revealed by Proteomic Analysis and Vaccination‐Induced Sterile Immunity,” PLoS Pathogens 9, no. 8 (2013): e1003492, 10.1371/journal.ppat.1003492. PMC3744408 23966853

[27] M. Osbourn , D. C. Soares , F. Vacca , et al., “HpARI Protein Secreted by a Helminth Parasite Suppresses Interleukin‐33,” Immunity 47, no. 4 (2017): 739–751.e5, 10.1016/j.immuni.2017.09.015. PMC5655542 29045903

[28] F. Colomb , A. Ogunkanbi , A. Jamwal , et al., “IL‐33‐Binding HpARI family Homologues With Divergent Effects in Suppressing or Enhancing Type 2 Immune Responses,” Infection and Immunity 92, no. 3 (2024): e0039523, 10.1128/iai.00395-23. PMC10929406 38294241

[29] F. Vacca , C. Chauché , A. Jamwal , et al., “A Helminth‐Derived Suppressor of ST2 Blocks Allergic Responses,” eLife 9 (2020): e54017, 10.7554/eLife.54017. PMC7234810 32420871

[30] A. Holgado , H. Braun , E. Van Nuffel , et al., “IL‐33trap is a Novel IL‐33–Neutralizing Biologic That Inhibits Allergic Airway Inflammation,” Journal of Allergy and Clinical Immunology 144, no. 1 (2019): 204–215, 10.1016/j.jaci.2019.02.028. PMC7610802 30876911

[31] K. Verstraete , F. Peelman , H. Braun , et al., “Structure and Antagonism of the Receptor Complex Mediated by human TSLP in Allergy and Asthma,” Nature Communications 8 (2017): 14937, 10.1038/ncomms14937. PMC5382266 28368013

[32] I. Markovic and S. N. Savvides , “Modulation of Signaling Mediated by TSLP and IL‐7 in Inflammation, Autoimmune Diseases and Cancer,” Frontiers in Immunology 11 (2020): 1557. 10.3389/fimmu.2020.01557 PMC7396566 32849527

[33] P. Bankhead , M. B. Loughrey , J. A. Fernández , et al., “QuPath: Open Source Software for Digital Pathology Image Analysis,” Scientific Reports 7, no. 1 (2017): 16878, 10.1038/s41598-017-17204-5. PMC5715110 29203879

[34] D. J. Smyth , S. H. Hodge , N. W. P. Ong , et al., “Vaccination Against helminth IL‐33 Modulators Permits Immune‐Mediated Parasite Ejection,” Cell Reports 44, no. 5 (2025): 115721, 10.1016/j.celrep.2025.115721. PMC7619366 40378045

[35] T. Liu , L. De , F. G. Santos , and S. H. Phan , “The Bleomycin Model of Pulmonary Fibrosis,” Methods in Molecular Biology 1627 (2017): 27–42, 10.1007/978-1-4939-7113-8_2. 28836192

[36] B. B. Moore and C. M. Hogaboam , “Murine Models of Pulmonary Fibrosis,” American Journal of Physiology‐Lung Cellular and Molecular Physiology 294, no. 2 (2008): L152–L160, 10.1152/ajplung.00313.2007. 17993587

[37] L. Blom , B. C. Poulsen , B. M. Jensen , A. Hansen , and L. K. Poulsen , “IL‐33 Induces IL‐9 Production in human CD4+ T Cells and Basophils,” PLoS ONE 6, no. 7 (2011): e21695, 10.1371/journal.pone.0021695. PMC3130774 21765905

[38] R. A. Rudders and G. T. Hensley , “Bleomycin Pulmonary Toxicity,” Chest 63, no. 4 (1973): 626–628, 10.1378/chest.63.4.626. 4121121

[39] H. Umezawa , “Studies on Bleomycin,” Taiwan Yi Xue Hui Za Zhi Journal of the Formosan Medical Association 68, no. 11 (1969): 569. 5266445

[40] H. Umezawa , “Chemistry and Mechanism of Action of Bleomycin,” Federation Proceedings 33, no. 11 (1974): 2296–2302. 4139043

[41] H. Umezawa , K. Maeda , T. Takeuchi , and Y. Okami , “New Antibiotics, Bleomycin A and B,” Journal of Antibiotics (Tokyo) 19, no. 5 (1966): 200–209. 5953301

[42] A. Yagoda , B. Mukherji , C. Young , et al., “Bleomycin, an Antitumor Antibiotic,” Annals of Internal Medicine 77, no. 6 (1972): 861–870, 10.7326/0003-4819-77-6-861. 4118759

[43] I. Barbayianni , I. Ninou , A. Tzouvelekis , and V. Aidinis , “Bleomycin Revisited: A Direct Comparison of the Intratracheal Micro‐Spraying and the Oropharyngeal Aspiration Routes of Bleomycin Administration in Mice,” Frontiers in Medicine 5 (2018): 269, 10.3389/fmed.2018.00269. PMC6165886 30320115

[44] C. Egger , C. Cannet , C. Gérard , et al., “Administration of Bleomycin via the Oropharyngeal Aspiration Route Leads to Sustained Lung Fibrosis in Mice and Rats as Quantified by UTE‐MRI and Histology,” PLoS ONE 8, no. 5 (2013): e63432, 10.1371/journal.pone.0063432. PMC3646779 23667616

[45] J. C. Gilhodes , Y. Julé , S. Kreuz , B. Stierstorfer , D. Stiller , and L. Wollin , “Quantification of Pulmonary Fibrosis in a Bleomycin Mouse Model Using Automated Histological Image Analysis,” PLoS ONE 12, no. 1 (2017): e0170561, 10.1371/journal.pone.0170561. PMC5249201 28107543

[46] H. C. Hawerkamp and P. G. Fallon , “Expelliarmus Helminthus! Harry Helminth and the Goblet of Alarmins,” Immunity 55, no. 4 (2022): 575–577, 10.1016/j.immuni.2022.03.014. 35417668

[47] F. Vacca and G. Le Gros , “Tissue‐Specific Immunity in Helminth Infections,” Mucosal Immunology 15, no. 6 (2022): 1212–1223, 10.1038/s41385-022-00531-w. PMC9178325 35680972

[48] N. J. Shubin , M. Clauson , K. Niino , et al., “Thymic Stromal Lymphopoietin Protects in a Model of Airway Damage and Inflammation via Regulation of Caspase‐1 Activity and Apoptosis Inhibition,” Mucosal Immunology 13, no. 4 (2020): 584–594, 10.1038/s41385-020-0271-0. PMC7312418 32103153

[49] R. Herro , R. Da Silva Antunes , A. R. Aguilera , K. Tamada , and M. Croft , “Tumor Necrosis Factor Superfamily 14 (LIGHT) Controls Thymic Stromal Lymphopoietin to Drive Pulmonary Fibrosis,” Journal of Allergy and Clinical Immunology 136, no. 3 (2015): 757–768, 10.1016/j.jaci.2014.12.1936. PMC4532661 25680454

