# Supplementary material for: Parasite‐Derived IL‐33 Inhibitors Prevent Bleomycin‐Induced Lung Fibrosis
Source: Eur J Immunol. 2026 Oct 8;56(10):e70291. doi: 10.1002/eji.70291 (PMC13647485; doi:10.1002/eji.70291)
Supplement: Supplementary file 1 [file EJI-56-e70291-s001.docx]

Supplementary Figure 1. HpARI2 and HpBARI_Hom2 administered in week 2 post-BLM cannot treat BLM-induced lung fibrosis**. A)** C57BL/6J mice were treated with 1 oropharyngeal challenge of 1.5 mg/Kg BLM under light isoflurane anaesthesia. HpARI2 and HpBARI_Hom2 (10μg of each protein), or vehicle control, were co-injected intraperitoneally at days 7, 9, and 11 post-BLM. **B)** Representative Masson’s Trichrome-stained sections of the lung. **C)** Representative images with overlay from HALO machine learning analysis whereby yellow is healthy tissue, green is fibrosis and red is airways and blood vessels. **D)** Blinded Ashcroft scoring of scanned Masson’s Trichrome-stained sections. **E)** Percentages of fibrotic tissue calculated via HALO machine learning analysis. Data are indicative of one experiment, which was pooled from 2 independent repeats to give total group sizes of n=10. Data are represented as mean ± SD. Statistical analysis was performed via One-Way ANOVA with Tukey’s multiple comparison test. *p<0.05, **p<0.01, ***p<0.001, ****p<0.0001.

 Supplementary Figure 2. Gating strategy to identify ILC2s and Th cells**. A)** Gating strategy for lung ILC2s and CD4+ Th cells. Total cells were selected and single cells were gated on FSC-A vs FSC-H. Single cells were gated on FSC-A versus live-dead viability stain to exclude debris and dead cells, then on CD45+ cells. Lineage−CD25+ cells were gated by CD25 versus Lineage and then CD4−ICOS+ ILC2s were gated by ICOS versus CD4. **B)** Lung cells in Figure 6F were analysed via flow cytometry and cells were stained to identify ICOS+ ILC2s: ICOS+CD25+CD4−Lineage−CD45+. Representative flow cytometry plots of ICOS versus CD4, on gated live CD25+Lineage−CD45+ cells.

Supplementary Figure 3. HpARI2 and HpBARI_Hom2 early treatment reduces lung cellular infiltration and negatively affects IL-33 signalling and activation on ILC2s**. A**) C57BL/6J mice were treated with 1.5 mg/Kg oropharyngeal BLM while a mix of HpARI2 and HpBARI_Hom2 (10μg each), or vehicle control, was injected intraperitoneally on days 0, 2, and 4. Mice were culled at day 7 post-BLM, and lung tissue was analysed via flow cytometry. **B)** Total lung cell counts were determined via hemacytometer count by two individuals. **C)** Lung cells were analysed via flow cytometry, and cells were stained to identify CD4^+^ T cells: CD4^+^Lineage^+^CD45^+^. Representative flow cytometry plots of CD4 versus lineage, on gated live CD45^+^ cells. CD4^+^Lineage^+^CD45^+^ T cell percentages **(D)** and numbers **(E)** of live cells. **F)** Lung cells were analysed via flow cytometry, and cells were stained to identify ICOS^+^ ILC2s: ICOS^+^CD25^+^CD4^−^Lineage^−^CD45^+^. Representative flow cytometry plots of CD25 versus lineage, on gated live CD45+ cells. Cells were then further gated cells on ICOS+CD4- (see **Supp Fig 1B**). **G)** ILC2 percentages of live cells and **H)** cell numbers. **I-J)** Representative plot of ST2 signal intensity and MFI on ILC2s. **K)** Median FSC-A indicating size of ILC2s. Data are indicative of one experiment, which was pooled from 2 independent repeats to give total group sizes of n=10. Data are represented as mean ± SD. Statistical analysis was performed via One-Way ANOVA with Tukey’s multiple comparison test. *p<0.05, **p<0.01, ***p<0.001, ****p<0.0001.

Supplementary Figure 4. HpARI2 and HpBARI_Hom2 negatively regulate eosinophil recruitment to the lung in response to BLM**. A)** Lung tissue of mice was analysed 7 days post-BLM administration. Lung cells were analysed via flow cytometry to identify total eosinophils: SiglecF^+^CD11c^−^CD45^+^. Representative flow cytometry plots of CD11c versus SiglecF, on gated live CD45^+^ cells. Percentages **(B)** and numbers **(C)** of live total SiglecF^+^CD11c^−^CD45^+^ eosinophils are reported. **D)** Lung cells were analysed via flow cytometry to identify neutrophils: Ly6G^+^CD11b^+^SiglecF^−^CD11c^−^CD45^+^. Representative flow cytometry plots of CD11b versus Ly6G, on gated live SiglecF^−^CD11c^−^CD45^+^ cells. Percentages **(E)** and numbers **(F)** of live Ly6G^+^CD11b^+^SiglecF^−^CD11c^−^CD45^+^ neutrophils are reported. Data are indicative of one experiment, which was pooled from 2 independent repeats to give total group sizes of n=10. Data are represented as mean ± SD. Statistical analysis was performed via One-Way ANOVA with Tukey’s multiple comparison test. *p<0.05, **p<0.01, ***p<0.001, ****p<0.0001.

Supplementary Figure 5. Gating strategy to identify neutrophils, eosinophils and alveolar macrophages. **A)** Gating strategy for lung and BAL eosinophils, macrophages, and neutrophils. Total cells were selected and single cells were gated on FSC-A vs FSC-H. Single cells were gated on FSC-A versus live-dead viability stain to exclude debris and dead cells, then on CD45+ cells. CD11c−SiglecF+ eosinophils, CD11c+SiglecF+ macrophages, and CD11c−SiglecF− monocytes were gated on CD11c versus SiglecF. CD11b+Ly6G+ neutrophils were further gated on CD11c−SiglecF− cells by CD11b versus Ly6G.
